# Silsesquioxane-Based Triphenylamine-Linked Fluorescent Porous Polymer for Dyes Adsorption and Nitro-Aromatics Detection

**DOI:** 10.3390/ma14143851

**Published:** 2021-07-09

**Authors:** Qingzheng Wang, Masafumi Unno, Hongzhi Liu

**Affiliations:** 1 Key Laboratory of Special Functional Aggregated Materials, School of Chemistry and Chemical Engineering, Ministry of Education Shandong University, 27 Shanda Nanlu, Jinan 250100, China; wangqingzheng@sdu.edu.cn; 2 Department of Chemistry and Chemical Biology, Graduate School of Science and Technology, Gunma University, Kiryu 376-8515, Japan; 3 Key Laboratory of Organosilicon and Materials Technology of the Ministry of Education, Hangzhou Normal University, Hangzhou 311121, China

**Keywords:** nanoporous polymer, silsesquioxane, triphenylamine, nitro-aromatics detection, dye adsorption

## Abstract

In order to enrich hybrid materials, a novel fluorescent silsesquioxane-based polymer (denoted as PCS-OTS) was synthesized by Friedel-Crafts reaction starting from octavinylsilsesquioxane (OVS) with triphenylamine-functionalized silsesquioxane monomer (denoted as OTS) with AlCl_3_ as catalyst. PCS-OTS possessed a high surface area of 816 m^2^/g and a unique bimodal pore structure. The triphenylamine unit endowed PCS-OTS with excellent luminescence, which made it act as a sensitive chemical sensor and detect p-nitrophenol with high sensitivity (K_SV_ = 81,230 M^−1^). Moreover, PCS-OTS can significantly remove dyes, and the respective adsorption capacity for Rhodamine B (RB), Congo red (CR) and Methyl Orange (MO) is 1935, 1420 and 155 mg/g. Additionally, it could simultaneously remove multiple dyes from water by simple filtration and be easily regenerated. This hybrid porous polymer can be a good choice for water treatment.

## 1. Introduction

Cage silsesquioxanes (SQs) are nano-sized hybrid molecules comprising an inorganic cage silica core and eight functional organic groups at the corners [1]. This multifunctional nature with rigid skeleton makes it an ideal building block to construct hybrid porous materials, especially those with high surface area and with good thermal, chemical and mechanical stability [2,3,4]. Due to their structure adjustability and high specific surface area, silsesquioxane-based porous polymers have been developed as a new type of porous materials, and recently they have been widely investigated for their application in gas storage and separation, environmental remediation, catalysis, sensors and so on [5].

The introduction of organic fluorescent units into silsesquioxane-based porous materials could endow materials with excellent luminescence owing to fluorescence quenching prevented by cages, which can be utilized to detect guest ions or molecules as chemical sensors. Nitro-aromatics (NACs) have been widely used in agricultural chemicals, synthesis and military applications. If used improperly, it could cause great harm to human health, public safety and ecological environment [6]. Therefore, it is highly demanded to develop efficient and robust sensors for detecting nitro-aromatics. For example, a silsesquioxane-based fluorescent porous polymer (HPP-1–HPP-3) was prepared by Heck reaction from octavinylsilsesquioxane (OVS) with ethene derivatives containing bis-, tri-, or tetrakis-bromophenyl groups and high selectivity for detecting 2,4,6-trinitrotoluene (TNT) and picric acid (PA) [5]. Triphenylamine (TPA) and its derivatives are important nitrogenous heterocyclic compounds, which have been proven to be another building block to produce porous polymers with high porosity and high fluorescence efficiency [7,8,9]. For example, triphenylamine-linked silsesquioxane-based porous polymers could be prepared via the Heck reaction or the Friedel-Crafts reaction [10]. Recently, the straightforward oxidative coupling polymerization was used to prepare hybrid porous polymer using triphenylamine functionalized silsesquioxane-based precursor (OTS) as a single precursor, which could detect nitro-aromatics and adsorb I_2_ [11]. As a new cage monomer, it is significant to develop new fluorescent porous polymer derived from OTS by different methods and explore their multiple applications.

As people’s requirements for printing are getting higher and higher, a large number of organic dyes are applied to textile and printing industries, which increases the pollution to the environment [5]. In order to curb the impacts of pollutants, wastewater needs to be treated before being discharged to the environment. Among wastewater treatment methods, adsorption has attracted intense interest as the easiest, most effective and economical technique [5]. Silsesquioxane-based porous materials have exhibited good adsorptions for dyes due to their high surface area and controllable pore size. For example, a silsesquioxane-based, thiophene-linked porous material (THPP) displayed a good adsorption capacity of 1402 mg/g for Rhodamine B (RB) and 862 mg/g for Methylene Blue (MB), respectively [5]. However, there still are some important issues to be addressed in this field and newer silsesquioxanes-based porous polymers are needed to meet the strict requirements of the environment.

**Scheme 1:**
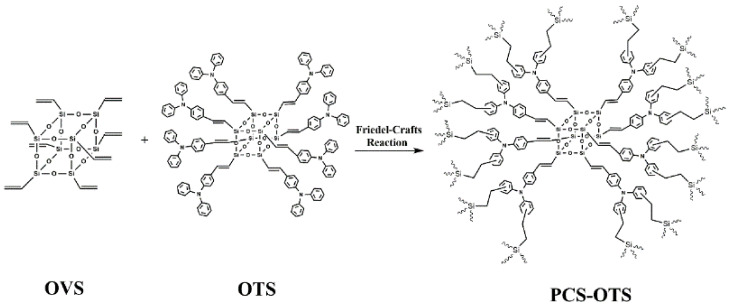
Synthesis route of PCS-OTS.

In this work, a novel fluorescent silsesquioxane-based polymer (PCS-OTS) was prepared by the Friedel-Crafts reaction from OVS with OTS (Scheme 1). The resulting porous polymer offers a high specific surface area, excellent luminescence and hierarchical porous structure. These excellent properties motivated us to investigate the porous material for multiple applications, namely, nitroaromatic explosives detection and dyes adsorption.

## 2. Materials and Methods

### 2.1. Materials

OVS and OTS were synthesized from previous reports [11]. Detailed synthesis information is provided in the Supplementary Material. Other reagents were purchased through commercial vendors. N,N-Dimethylformamide (DMF, AR), Triethylamine (Et_3_N, AR), 1,2-dichloroethane (AR), Tetrahydrofuran (THF, AR), Chloroform (AR), Methanol (AR), Acetone (AR), Calcium hydride (CaH_2_), Anhydrous aluminum chloride, 1,3-dinitrobenzene (1,3-DTB), p-nitroaniline (PNA), 2,4-dinitrotoluene (2,4-DNT), p-nitrotoluene (PNT), 4-nitrochlorobenzene (4-NCB), 4-nitrophthalic acid (4-NPA), p-nitrophenol (PNP) and o-nitrophenol (ONP) were purchased from Sinopharm Chemical Reagent Co., Ltd. (Shanghai, China). Rhodamine B (RB), Congo red (CR) and Methyl orange (MO) were purchased from Shanghai Macklin Biochemical Co., Ltd. (Shanghai, China). DMF was dried over CaH_2_ at 80 °C for 12 h and distilled under vacuum, then stored with 4 Å molecular sieves prior to use. Triethylamine (Et_3_N) and chloroform were dried over CaH_2_ and used freshly. 1,2-dichloroethane was dried by distillation over calcium hydride under reflux prior to use.

### 2.2. Material Characterization and Adsorption Experiments

Fourier-transform infrared (FTIR) spectra were recorded with a Bruker Tensor 27 spectrophotometer (Bruker, Bremen, Germany) with a disc of KBr from 4000 to 400 cm^−1^ at a resolution of 4 cm^−1^. UV–vis spectra were determined by Hitrachi spectrophotomer (HITACHI, Tokyo, Japan). Fluorescence spectra were recorded through luminescence spectrophotometer Hitrachi F-7000 FL (HITACHI, Tokyo, Japan). Solid-state ^13^C cross-polarization/magic-angle-spinning (CP/MAS) NMR and ^29^Si MAS NMR spectra were performed using a Bruker Avance-500 NMR spectrometer (Bruker, Zurich, Switzerland) operating at a magnetic field strength of 9.4 T. Powder X-ray diffraction (PXRD) was measured using a Riguku D/MAX 2550 diffractometer (Shimadzu, Tokyo, Japan) under Cu-Kα radiation, 40 kV, 200 mA with a 2θ range of 5–80° (scanning rate of 10° min^−1^) at room temperature. Thermogravimetric Analyses (TGA) were conducted with a Mettler-Toledo SDTA-854 TGA system (Mettler-Toledo, Zurich, Switzerland) in nitrogen (100 mL min^−1^) at a heating rate of 10 °C min^−1^ from 30 to 800 °C. Elemental analyses were performed using an Elementar EL III elemental analyzer (Elementar, Vario EL Cube, Langenselbold, Germany). High-resolution transmission electron microscopy (HR-TEM) experiments were recorded using a JEM 2100 electron microscope (JEOL, Tokyo, Japan) with an acceleration voltage of 200 kV. Field-emission scanning electron microscopy (FE-SEM) experiments were recorded with a HITACHI S4800 spectrometer (HITACHI, Tokyo, Japan). Nitrogen adsorption-desorption isotherm measurements were performed with a Quadra Sorb SI apparatus (Quantachrome Instruments, Boynton Beach, FL, USA) at 77 K. Nonlocal density functional theory (NL-DFT) pore-size distributions were confirmed by using the carbon/slit-cylindrical pore mode of the Quadrawin software (Quantachrome Instruments, Boynton Beach, FL, USA).

Adsorption experiments are provided in the Supplementary Material.

### 2.3. Synthesis of Hybrid Luminescent Porous Polymer (PCS-OTS)

OVS (0.632 g, 1 mmol), OTS (1.283 g, 0.5 mmol), anhydrous aluminum chloride (0.534 g, 4 mmol) and 1,2-dichloroethane (25 mL) were charged in an oven-dried flask. The mixture was stirred at room temperature for 0.5 h and then refluxed for 24 h. After cooling to room temperature, the mixture was filtered and washed with THF, methanol, acetone and chloroform, sequentially, in order to remove any unreacted monomers or remained catalyst. The product was further purified under the Soxhlet extractor (Sichuan Shubo Co., Ltd., Sichuan, China) with THF for 24 h and methanol for 24 h, and then dried under vacuum (Jinan Hainuo Co., Ltd., Jinan, Shandong, China) at 80 °C for 24 h to afford a brown solid (1.90 g). Yield: 99%.

## 3. Results and Discussion

### 3.1. Preparation and Characterization

In order to prepare the porous polymer, the Friedel-Crafts reaction was performed from OVS and OTS (Scheme 1). The FTIR spectrum of PCS-OTS (Figure S1) shows a characteristic band of C–N stretching vibration at 1272 cm^−1^. At 1411, 1535 and 1657 cm^−1^, C=C vibrational modes of vinyl and phenyl groups appeared. A strong band at 1118 cm^–1^ belongs to the stretching vibration of Si–O–Si groups for OVS, OTS and PCS-OTS. A new band at 2935 cm^−1^ was attributed to the stretching vibration of CH_2_ groups, which are formed by the Friedel-Crafts reaction.

**Figure 1:**
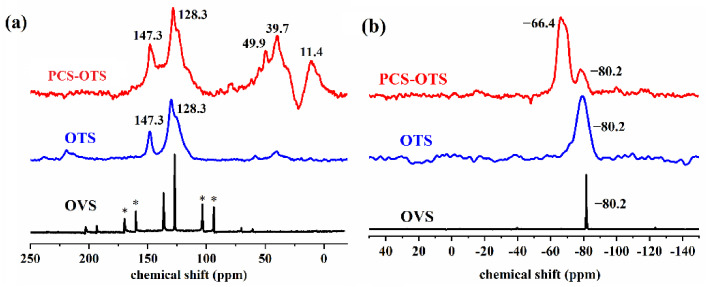
(**a**) Solid state ^13^C CP/MAS NMR spectra of OVS, OTS, and PCS-OTS (* represents spinning sidebands). (**b**) Solid state ^29^Si MAS NMR spectra of OVS, OTS and PCS-OTS.

Figure 1a shows the solid-state ^13^C MAS NMR spectrum of OTS and PCS-OTS. The signals located at 128.3 and 147.3 ppm from PCS-OTS can be treated as a result of substituted phenyl carbons linked to the nitrogen atom and unsubstituted phenyl carbons, and two peaks of ethenylene carbons (SiCH=CH, SiCH=CH) overlapped with the above-mentioned peaks, respectively [5]. In addition, the signals in the aliphatic region 5–60 ppm are assigned to Si–CH_2_–CH_2_- or Si–CH(CH_3_)-groups formed by Friedel-Crafts reaction [12]. The Solid-state ^29^Si NMR spectra of OTS and PCS-OTS are shown in Figure 1b. Relative to a single peak at −80.2 ppm in the solid ^29^Si MAS NMR spectrum of OTS, another peak at d = −66.4 ppm is observed, which is attributed to T_3_ silicons connected with ethylene. The peak at −80.2 ppm is the overlap of T_3_ silicons connected to unreacted vinyl groups and OTS [5].

Thus, the results of FTIR, ^13^C and ^29^Si NMR, and elemental analysis (Table S1) clearly showed that OVS completely reacted with OTS to produce PCS-OTS by facile Friedel-Crafts reaction.

### 3.2. Porosity

**Figure 2:**
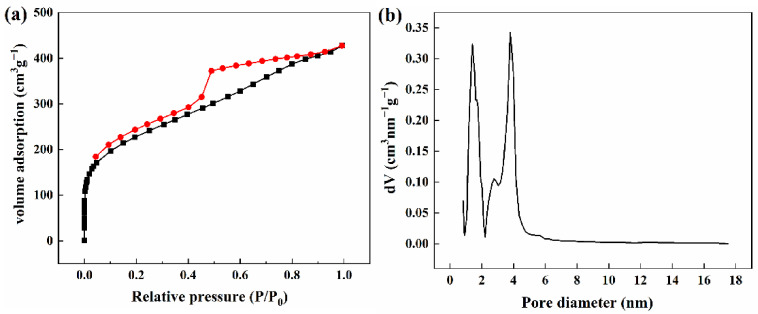
(**a**) N_2_ adsorption-desorption isotherm of PCS-OTS (Red and black line denote adsorption and desorption branches). (**b**) Pore size distribution curve of PCS-OTS calculated by NL-DFT.

Nitrogen adsorption-desorption isotherm was measured at 77 K, to study the porosity of PCS-OTS. It showed a rapid uptake increase at a low relative pressure (P/P_0_ = 0–0.01), and then gradual increase at a high pressure. Obvious hysteresis was observed as seen in Figure 2a, indicating that it contained both micropores and mesopores [5]. The PCS-OTS possess high specific surface area (S_BET_ = 816 m^2^/g), and the t-plot method was used to calculate the micropore surface area, which was 289 m^2^/g. In Figure 2b, pore size distribution was conformed from the nonlocal density functional theory (NL-DFT), which demonstrated unique bimodal structure with mesopores at 3.8 nm and the uniform micropores at 1.4 nm [13]. The porosity results of PCS-OTS are shown in Table S2.

### 3.3. Thermal Property and Morphology

Thermogravimetric Analysis (TGA) was performed under N_2_ at 10 °C min^−1^ to evaluate the thermal stability of PCS-OTS. The decomposition temperatures Td_5%_ of OTS and PCS-OTS were 264 and 370 °C, respectively (Figure S2). This indicates that the formation of highly cross-linked network makes PCS-OTS more stable than OTS. Powder X-ray diffraction (PXRD) analysis showed that PCS-OTS possessed an amorphous feature with a broad diffraction peak at ≈22° (2θ), assigned to Si–O–Si bonded units (Figure S3) [14,15,16]. The random cross-linking method makes the materials an amorphous structure.

**Figure 3:**
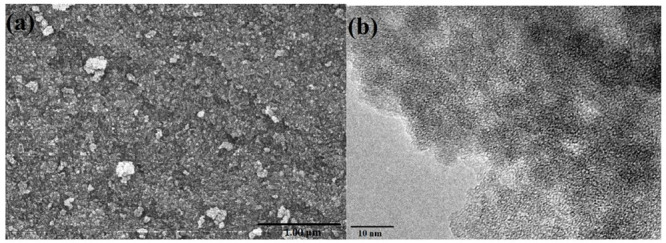
(**a**) FE-SEM image and (**b**) HR-TEM image of PCS-OTS.

The field emission scanning electron microscopy (FE-SEM) reveals that PCS-OTS consists of agglomerated particles (Figure 3a). Partially ordered porosity can also be observed from the high-resolution transmission electron microscope (HR-TEM) image, probably due to the ordered arrangement of organic linkers (Figure 3b).

### 3.4. Photophysical Properties

**Figure 4:**
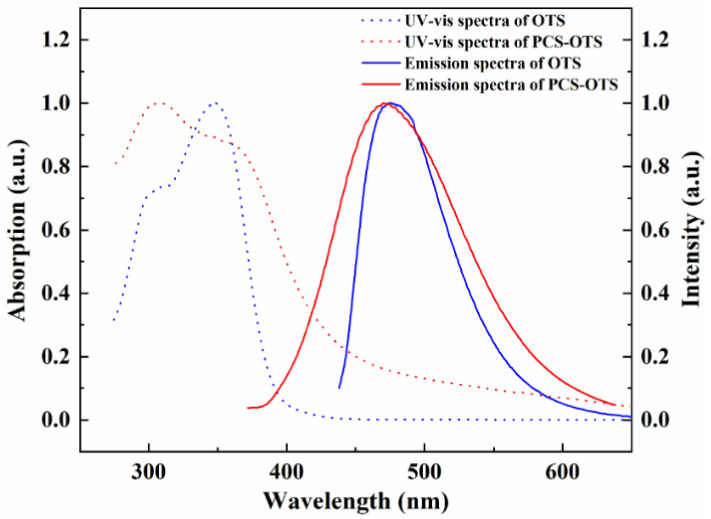
UV-vis absorption and emission spectra of PCS-OTS suspension (0.3 mg/mL) and OTS (10^−6^ M) in DMF (λ_ex_ = 350 nm).

The photophysical properties of PCS-OTS suspension (0.3 mg/mL) and OTS (10^−6^ M) in DMF solution were studied by UV-vis absorption and fluorescence emission spectroscopy (Figure 4). As expected, PCS-OTS and OTS similarly showed absorption from 300 to 350 nm and absorption maxima were observed at 350 and 306 nm, respectively. These peaks can be attributed to the π–π* transitions of phenyl groups [17,18]. In addition, due to the successful incorporation of triphenylamine units into the cross-linking network, they both similarly exhibit fluorescence with the maxima at 475 nm.

### 3.5. Detection of Nitro-Aromatics by PCS-OTS Suspension

**Figure 5:**
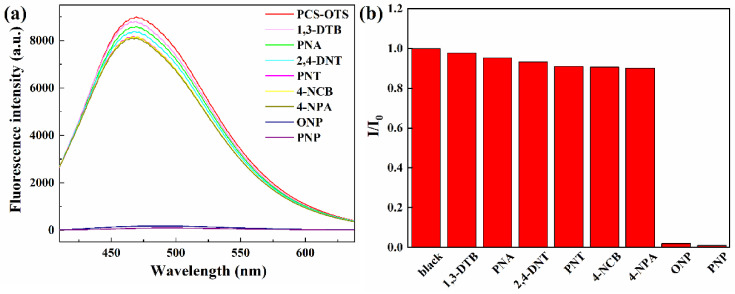
(**a**) Fluorescence emissions of PCS-OTS suspension in DMF (0.3 mg/mL) upon addition of nitro-aromatics, including 1,3-DTB, PNA, 2,4-DNT, PNT, 4-NCB, ONP and PNP (1 × 10^−3^ M, λ_ex_ = 350 nm). (**b**) Changes in relative emission intensities (I/I_0_) of PCS-OTS.

The fluorescence quenching effect of different nitro-aromatics on PCS-OTS suspensions (0.3 mg/mL in DMF) was investigated by a comparison in the changes of the fluorescence emission spectra of PCS-OTS. The fluorescence of PCS-OTS suspension was markedly quenched upon addition of p-nitrophenol (PNP) and o-nitrophenol (ONP), whereas other nitro-aromatics including 1,3-dinitrobenzene (1,3-DTB), p-nitroaniline (PNA), 2,4-dinitrotoluene (2,4-DNT), p-nitrotoluene (PNT), 4-nitrochlorobenzene (4-NCB), and 4-nitrophthalic acid (4-NPA) had nearly no effect (Figure 5a). As shown in Figure 5b, a considerably high quenching efficiency was observed for ONP (98%) and PNP (99%), respectively.

**Figure 6:**
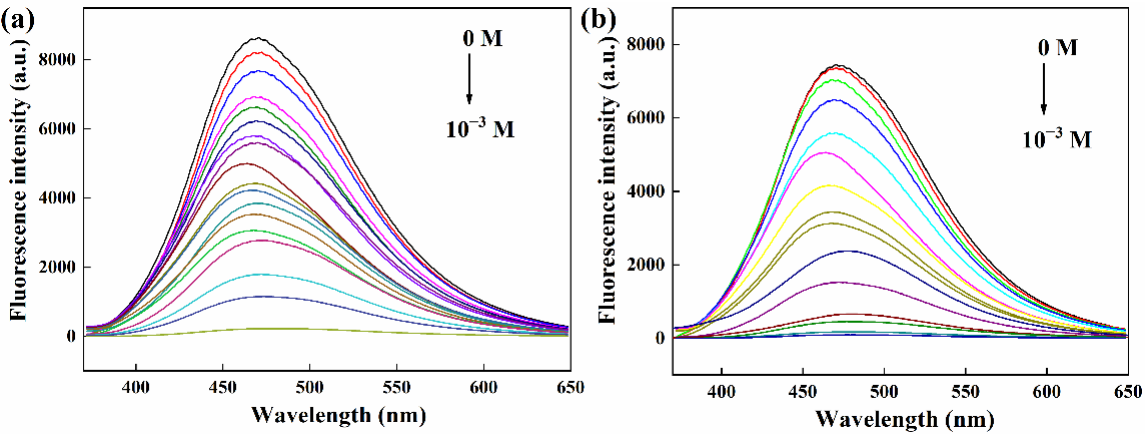
Fluorescence spectra of PCS-OTS suspension in DMF (0.3 mg/mL) with various concentrations of (**a**) ONP and (**b**) PNP (0 to 1 × 10^−3^ mol/L, λ_ex_ = 350 nm).

A fluorescence titration was performed to obtain the sensing behavior of PCS-OTS for nitro-aromatics. As shown in Figure 6a,b with the incremental addition of ONP and PNP, the fluorescence intensity of PCS-OTS suspension gradually decreases. Using Stern-Volmer equation, quenching efficiency was calculated (see details in the Supporting Materials). The largest quenching constants of ONP and PNP are 32,976 and 81,230 M^−1^, respectively (Figure S4).

The higher quenching efficiencies of PCS-OTS was observed for PNP than ONP. The quenching mechanism of PCS-OTS for ONP and PNP can be explained by fluorescence resonance energy transfer (FRET) [6,19,20,21]. As shown in Figure S5, a spectral overlap was observed for the absorption spectra of ONP and PNP and the emission spectrum of PCS-OTS in the range of 400–500 nm. For nitro-aromatics, PNP has the greatest overlap, followed by ONP, while there was negligible spectral overlap for other nitro-aromatics 1,3-DTB, PNA, 2,4-DNT, PNT and 4-NCB. The highest quenching efficiency of PCS-OTS to PNP compared with other NACs was explained by the result of maximum overlap of the absorption spectrum of PNP with the emission band of PCS-OTS. These results demonstrated the high selectivity and discriminative sensing ability of PCS-OTS for phenolic-nitroaromatics over other interfering non-phenolic analytes.

### 3.6. Adsorption for Dyes

Most dyes will destroy the transparency of waterbody, consume a large amount of oxygen in the water, affect the growth of aquatic organisms and microorganisms and destroy the self-purification of waterbody. In addition, dyes have teratogenic and carcinogenic effects, causing serious damage to the human health [22,23,24,25]. Thus, it is crucial to find a suitable material to remove dyes rapidly and conveniently from the wastewater. Significantly, PCS-OTS has a large specific surface area and porous properties, so the dye adsorption of PCS-OTS was further investigated. Three dyes, such as Rhodamine B (RB), Congo red (CR) and Methyl orange (MO) are chosen to investigate the adsorption capability of this porous polymer.

**Figure 7:**
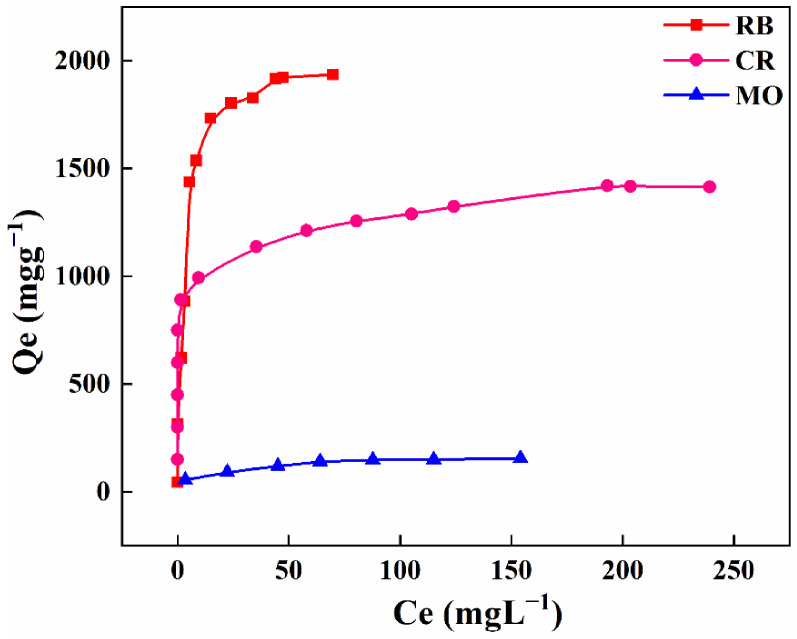
Equilibrium adsorption isotherms of dyes (C_e_ is the post-adsorption concentrations of solutions).

PCS-OTS (3 mg) was added to 20 mL of dye aqueous solutions with different concentrations. To research the adsorption kinetics of PCS-OTS, an UV-vis spectrophotometer was performed to gauge the changes in intensities as a function of concentration of dyes. As shown in Figure 7, the maximum equilibrium adsorption capacity (Q_e_) of RB, CR and MO is 1935, 1420 and 155 mg/g, respectively. Compared with related porous materials (HPP-3: 1666 mg/g and PFCMP-0: 1376 mg/g), PCS-OTS showed significantly higher adsorption capacities for RB and CR (Table S3). The Q_e_ of PCS-OTS for RB, CR and MO showed the order of RB > CR > MO. Table S4 shows the physical and chemical properties of the dyes [26,27,28]. By comparison, PCS-OTS has a large adsorption capacity for dyes of larger sizes. This phenomenon can be attributed to the fact that the polymer has unique micropores and mesopores. Larger size dyes, i.e., RB and CR, can enter and stay in the mesopores or clog micropores, while smaller dye molecules such as MO could easily pass through the pores [27,29,30]. Although the size of CR is larger than RB, its adsorption capacity is higher than RB. This is mainly due to the oxygen atoms and TPA unit, which make PCS-OTS more electron-rich. Therefore, PCS-OTS is prone to adsorb cationic dye of RB through electrostatic interaction [31]. In summary, the properties of dyes and absorbents, such as the type and size of dyes, the pore structure parameters and chemical structure of absorbents all affect the adsorption behavior [26,32].

Langmuir and Freundlich isotherm models are used to fit the equilibrium data of dye adsorption in order to investigate the adsorption mechanism of PCS-OTS (Figure S6) [33,34]. Table S4 shows the fitting isotherm parameters. The correlation coefficients of the Langmuir isotherm model (R_L_^2^, 0.997 for RB, 0.997 for CR and 0.993 for MO) were higher than those simulated by the Freundlich isotherm models (R_F_^2^, 0.816 for RB, 0.979 for CR and 0.979 for MO). The results indicated that the adsorption behavior of PCS-OTS on dyes is a well fit with the Langmuir isotherm model and certify monolayer adsorption of process. The theoretical maximum adsorption capacities (Q_m_) for RB, CR and MO on PCS-OTS are calculated as 1935, 1420 and 155 mg/g, respectively, for the corresponding adsorption models. 

Considering the highest Q_max_ of RB, a fixed initial concentration of 30 mg/L was selected to investigate adsorption kinetics. As shown in Figure S7, the adsorption rate was fast, and more than 95% RB was adsorbed after 50 min, which accounted for the fast adsorption kinetics of this porous polymer. The value of R^2^ demonstrates that the pseudo-second-order kinetic model is more suitable for explaining this adsorption process (Table S6 and Figure S8) [26,29].

Another adsorption experiment was performed to demonstrate that it could simultaneously remove the mixed dyes in wastewater. Their higher Q_max_, RB and CR were used to prepare simulated wastewater. PCS-OTS (0.2 g) was used to fill a 2 cm high filter bed in a slim glass column. A mixture solution, in which the concentration of dyes (CR and RB) was 20 mg/L, was used for evaluation. As shown in Figure S9, after the filtration, colorless solution and UV-vis spectrophotometer results indicate that dyes were removed completely. These results revealed their strong ability to deeply purify wastewater containing complex pollutants.

**Figure 8:**
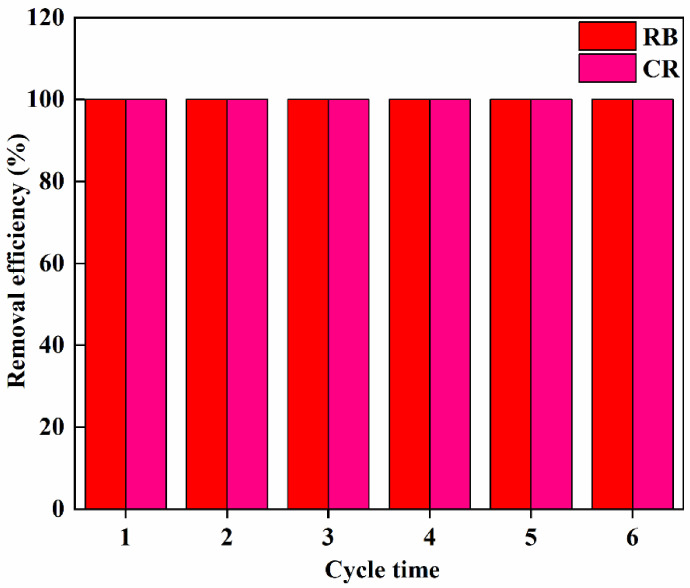
Recyclability of PCS-OTS for dyes adsorption.

To inquire into the renewable performance of adsorbents, cyclic adsorption-regeneration experiments were put into effect. We chose a mixed dye-water solution of RB and CR for adsorption. The polymer after adsorbing the dye is washed with hot methanol for desorption. Significantly, the removal efficiency of PCS-OTS for RB and CR remains nearly 100% after six cycles (Figure 8). The conclusion is that PCS-OTS can be reused to remove dyes.

## 4. Conclusions

In summary, OVS and OTS reacted by the Friedel-Crafts reaction to synthesize a new silsesquioxane-based triphenylamine-linked porous polymer (PCS-OTS). The PCS-OTS exhibits a high surface area of 816 m^2^/g and a good thermal stability as well as excellent luminescence properties and acts as a sensitive chemical sensor to detect nitro-aromatics with high sensitivity toward p-nitrophenol (PNP) and o-nitrophenol (ONP). Furthermore, it also exhibited a high adsorption capacity of Rhodamine B (1935 mg/g), Congo red (1420 mg/g) and Methyl orange (155 mg/g), respectively. More importantly, multiple pollutants in wastewater can be simultaneously removed by simple filtration and the adsorbent is easily recycled. This hybrid porous polymer can be very promising for applications in water treatment.

## Data Availability

Data are contained within the article.

## Supplementary Materials

The following are available online at https://www.mdpi.com/article/10.3390/ma14143851/s1, Figure S1: FTIR spectra of OVS, OTS and PCS-OTS, Figure S2: TGA curves of OVS, OTS and PCS-OTS in N_2_, Figure S3: Powder XRD patterns of PCS-OTS, OTS and OVS, Figure S4: Stern-Volmer plot of I_0_/I-1 of PCS-OTS versus [ONP] (a) and [PNP] (b) and K_SV_ value, Figure S5: UV-vis absorption spectra of various analytes in DMF, and UV-vis and emission spectra of PCS-OTS in DMF suspension, Figure S6: The Langmuir (a) and Freundlich (b) isotherm models for dyes solution onto PCS-OTS, Figure S7: (a)Effect of contact time on the adsorption of Rhodamine B by PCS-OTS (inset is the photo of the dyes at 0 min and 240 min). (b) Removal efficiency of Rhodamine B on PCS-OTS, Figure S8: The pseudo-first-order (a) and pseudo-second-order (b) kinetic model plots for RB adsorption by the PCS-OTS, Figure S9: Process for the purification of the simulated wastewater, Table S1: Elemental analysis of OTS and PCS-OTS, Table S2: Porosity data of PCS-OTS, Table S3: Comparison of adsorbents of removal of Rhodamine B and Congo red, Table S4: Physical and chemical properties of dyes, Table S5: Summary of Langmuir and Freundlich isotherm model parameters for the absorption of dyes, Table S6: Kinetic parameters of RB onto adsorbents, Scheme S1: Synthesis route of OTS.
