# Supplementary material for: Silsesquioxane-Based Triphenylamine-Linked Fluorescent Porous Polymer for Dyes Adsorption and Nitro-Aromatics Detection
Source: Materials (Basel). 2021 Jul 9;14(14):3851. doi: 10.3390/ma14143851 (PMC8306194; doi:10.3390/ma14143851)
Supplement: Supplementary file 1 [file materials-14-03851-s001.zip › materials-1273770- for Publication SM.pdf]

materials

Supplementary Material

Silsesquioxane-Based Triphenylamine-Linked Fluorescent Porous Polymer for Dyes Adsorption and Nitro-Aromatics Detection

Qingzheng Wang [1,2], Masafumi Unno [2,*] and Hongzhi Liu [1,3,*]

[1] Key Laboratory of Special Functional Aggregated Materials, School of Chemistry and Chemical Engineering, Ministry of Education Shandong University, 27 Shanda Nanlu, Jinan 250100, China; wangqingzheng@sdu.edu.cn (Q.W.).
[2] Department of Chemistry and Chemical Biology, Graduate School of Science and Technology, Gunma University, Kiryu 376-8515, Japan
[3] Key Laboratory of Organosilicon and Materials Technology of the Ministry of Education, Hangzhou Normal University, Hangzhou 31112, China
* Correspondence: unno@gunma-u.ac.jp (M.U.); liuhongzhi@sdu.edu.cn (H.L.)

Table of Contents

Table S1 Elemental analysis of OTS and PCS-OTS.
Table S2 Porosity data of PCS-OTS.
Table S3 Comparison of adsorbents of removal of Rhodamine B and Congo red.
Table S4 Physical and chemical properties of dyes.
Table S5 Summary of Langmuir and Freundlich isotherm model parameters for the absorption of dyes.
Table S6 Kinetic parameters of RB onto adsorbents.
Figure S1. FTIR spectra ofOVS, OTS and PCS-OTS.
Figure S2. TGA curves of OVS, OTS and PCS-OTS in N_2.
Figure S3. Powder XRD patterns of PCS-OTS, OTS and OVS.
Figure S4. Stern-Volmer plot of $I_0/I{-}1$ of PCS-OTS versus [ONP] (a) and [PNP] (b) and K_{SV} value.
Figure S5. UV-vis absorption spectra of various analytes in DMF, and UV-vis and emission spectra of PCS-OTS in DMF suspension.
Figure S6. The Langmuir (a) and Freundlich (b) isotherm models for dyes solution onto PCS-OTS.
Figure S7. (a)Effect of contact time on the adsorption of Rhodamine B by PCS-OTS (inset is the photo of the dyes at 0 and 240 min). (b) Removal efficiency of Rhodamine B on PCS-OTS.
Figure S8. The pseudo-frist-order (a) and pseudo-second-order (b) kinetic model plots for RB adsorption by the PCS-OTS.
Figure S9. Process for the purification of the simulated wastewater.

Materials and Methods

1. Synthesis of octavinylsilsesquioxane (OVS)

Octavinylsilsesquioxane (OVS) was synthesized from the previous reports [1].

2. Synthesis of Cage Monomer (OTS)

The precursor, triphenylamine-functionalized silsesquioxane monomer (OTS), was synthesized according to our previous report [1].

In an oven-dried flask was added OVS (758.4 mg, 1.2 mmol), (4-Bromophenyl)diphenylamine (TPA-Br, 4.67 g, 14.4 mmol), palladium acetate (108 mg, 0.48 mmol) and tris(2-

methylphenyl) phosphine (0.3 mg, 0.96 mmol) in DMF/Et_3N (30 mL/10 mL) under argon. The mixture was stirred for 30 min at room temperature and subsequently heated at 100 °C for 72 h. After cooling to room temperature, the mixture was filtered and washed with tetrahydrofuran. The product was obtained by rotary evaporation, and purified by column chromatography (silica gel, petroleum ether: dichloromethane = 1:2). The product was dried under vacuum at 70 °C for 24 h. OTS was obtained as a yellow powder (1.238 g). Yield: 40%.

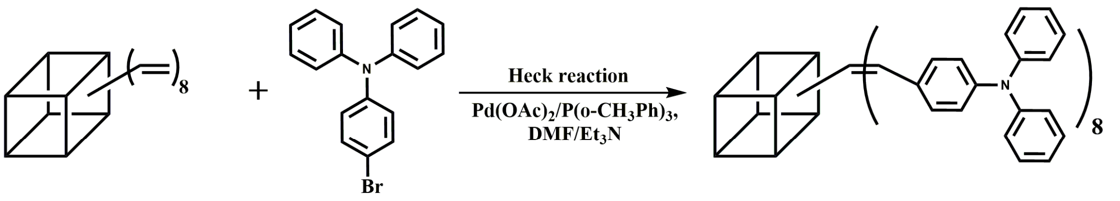

Scheme S1. Synthesis route of OTS.

3. Synthesis of Hybrid Luminescent Porous Polymer (PCS-OTS)

OVS (0.632 g, 1 mmol), OTS (1.283 g 0.5 mmol), anhydrous aluminum chloride (0.534 g, 4 mmol) and 1,2-dichloroethane (25 mL) were charged in an oven-dried flask. The mixture was stirred at room temperature for 0.5 h and then refluxed for 24 h. After cooling to room temperature, the mixture was filtered and washed with THF, methanol, acetone, chloro-form, sequentially, in order to remove any unreacted monomers or residuary catalyst. The product was further purified under the Soxhlet extractor with THF for 24 h and methanol for 24 h, respectively, and then dried under vacuum at 80 °C for 24 h to obtain a brown solid (1.90 g). Yield: 99%.

4. Stern–Volmer Equation

$$I_0/I = K_{SV}\,[Q] + 1 \quad (1)$$

I_0 is the initial intensity of the PCS-OTS suspension and I is the intensity with a given concentration of nitro-aromatics. [Q] is the molar concentration of nitro-aromatics and K_{SV} is the quenching constant.

5. Adsorption Experiments

The dye adsorption experiments were done by taking three dye molecules as adsorbates, including Rhodamine B (RB), Congo Red (CR), and Methyl Orange (MO). Dye solutions were prepared by deionized water with known amounts of RB, CR, and MO. In the process of dye removal, 3 mg adsorbents were placed into to 20 mL dye solutions of different initial concentrations and the mixture was stirred at room temperature for 24 h. The concentration of dye solutions before and after adsorption was determined by employing a UV-vis spectrophotometer (the maximum absorption wavelengths for CR, RB and MO are 497, 554 and 466 nm, respectively).

The amount of adsorbate adsorbed at equilibrium Q_e (mg/g) was calculated from the Equation (2):

$$Q_e = \frac{(C_0 - C_e) \times V}{m} \quad (2)$$

where m (g) represents the mass of dry adsorbent and V (L) is the volume of solution. C_0 and C_e (mg/L) are the initial and final (post-adsorption) concentrations of solutions, respectively.

In the adsorption kinetic experiments, the adsorption capacity at time t (min) can be calculated using the Equation (3)

$$Q_t = \frac{(C_0 - C_t) \times V}{m} \tag{3}$$

Adsorption isotherms and relevant parameters are usually used to determine the adsorption mechanism. The obtained equilibrium adsorption data can be analyzed by fitting against the Langmuir (4) and Freundlich (5) isotherm models,

$$\frac{C_e}{Q_e} = \frac{C_e}{Q_m} + \frac{1}{K_L \times Q_m} \tag{4}$$

$$\ln Q_e = \ln K_F + \frac{1}{n} \times \ln C_e \tag{5}$$

where K_L and Q_m (mg/g) represent Langmuir constant and the maximum adsorption capacity calculated by Langmuir model, respectively. K_F and 1/n are Freundlich constants that describe the adsorption capacity and intensity, respectively.

Adsorption kinetic are of great importance in the adsorption process as they can predict the removal rate of certain pollutants from solutions and help get a better understanding of the adsorption mechanism.

The pseudo-first-order and pseudo-second-order models were used to simulate the adsorption kinetics in this research.

The linear from of pseudo-first-order can be expressed as the Equation (6):

$$\ln(q_e - q_t) = \ln q_e - k_1 \times t \tag{6}$$

The linear from of pseudo-second-order can be expressed as the Equation (7):

$$\frac{t}{q_t} = \frac{1}{k_2 \times {q_e}^2} + \frac{t}{q_e} \tag{7}$$

where q_e and qt refer to the adsorption capacity at equilibrium and at time t (min); k_1 (min^{-1}) and k_2 (g mg^{-1} min^{-1}) are the adsorption rate constants for the pseudo-first-order and pseudo-second-order, respectively.

Table S1. Elemental analysis of OTS and PCS-OTS.

**Sample**	**Experimental Value**		**Theoretical Value**	
	**C%**	**N%**	**C%**	**N%**
OTS	72.62	4.50	74.50	4.34
PCS-OTS	48.72	1.70	49.37	1.72

Table S2. Porosity data of PCS-OTS.

**S_{BET}[1] (m^2 g^{-1})**	**S_{micro}[2] (m^2 g^{-1})**	**V_{total}[3] (cm^3 g^{-1})**	**V_{micro}[4] (cm^3 g^{-1})**	**V_{micro}/V_{total}**
815.922	289.056	0.591	0.128	0.22

[1]—Surface area calculated from the N_2 isotherm. [2]—Microporous surface area calculated from the N_2 adsorption isotherm using the t-plot method. [3]—Total pore volume calculated at $P/P_0 = 0.99$. [4]—The micropore volume derived using the t-plot method.

Table S3. Comparison of adsorbents of removal of Rhodamine B and Congo red.

Adsorbate	Adsorbent	Physical Parameter ($m^2\ g^{-1}$)	Adsorption Capacity ($mg\ g^{-1}$)	References
RB	Fe_3O_4@POSS-SH	S_{BET}: 224.20	142.05	[2]
	MPSC/C	S_{BET}: 2580	785	[3]
	HP-TPP-3	S_{BET}: 1105	674.6	[4]
	Graphene sponge	S_{BET}: 399	72.5	[5]
	SO_3H-HSM	S_{BET}: 345.69	271	[6]
	Nanoporous THPP	S_{BET}: 915	1402	[7]
	HPP-3	S_{BET}: 1910	1666	[8]
	PCS-OTS	S_{BET}: 816	1935	This work
CR	PFCMP-0	S_{BET}: 901	1376.7	[9]
	CNT/MMO nanocompositeses	S_{BET}: 148	1250	[10]
	α-FeOOH Hollowspheres	S_{BET}: 96.9	275	[11]
	HPP-3	S_{BET}: 1910	1040	[8]
	Carbon nanotubes	S_{BET}: 789	882	[12]
	Porous BN nanosheets	S_{BET}: 1427	782	[13]
	PCS-OTS	S_{BET}: 816	1420	This work

Table S4. Physical and chemical properties of dyes.

Dyes	Molecular Structure	Molecular Size (nm^3)	Molecualr Weight (g/mol)	Nature	Adorption Wavelength (nm)
CR	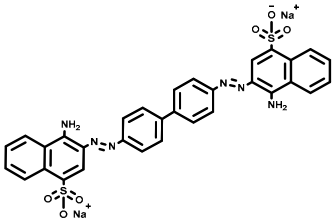	2.62 × 0.74 × 0.43	696	anionic	497
RB	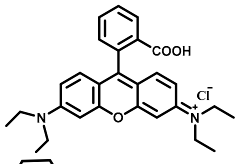	1.59 × 1.18 × 0.56	478	cationic	554
MO	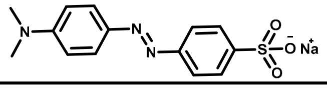	1.31 × 0.55 × 0.18	327	anionic	464

Table S5. Summary of Langmuir and Freundlich isotherm model parameters for the absorption of dyes.

Dyes	Langmuir Constants			Freundlich Constants		
	Q_m ($mg\ g^{-1}$)	K_L	R_L^2	K_F ($L\ mg^{-1}$)	n	R_F^2
RB	2000	0.472	0.997	701.53	3.60	0.816
CR	1428.57	0.294	0.997	823.37	10.18	0.979
MO	168.07	0.073	0.993	38.10	3.41	0.979

Table S6. Kinetic parameters of RB onto adsorbents.

Modal	Parameters	
Pseudo-first order	$Q_{e,cal}$ (mg/g)	69.8
	k_1 (h^{-1})	0.0411
	R^2	0.781
Pseudo-second order	$Q_{e,cal}$ (mg g^{-1})	183.5
	k_2 (g mg^{-1} h^{-1})	0.0015
	R^2	0.999

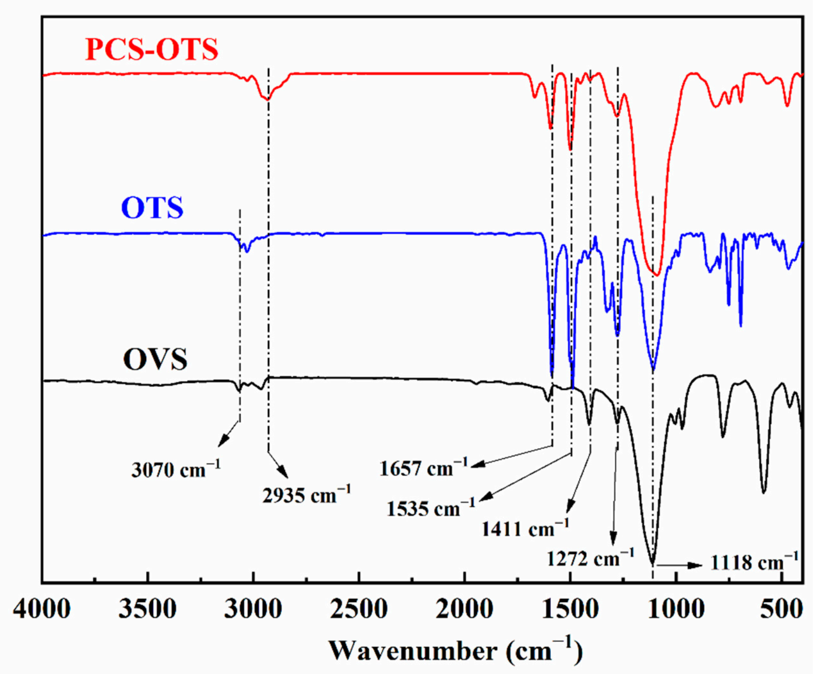

Figure S1. FTIR spectra of OVS, OTS and PCS-OTS.

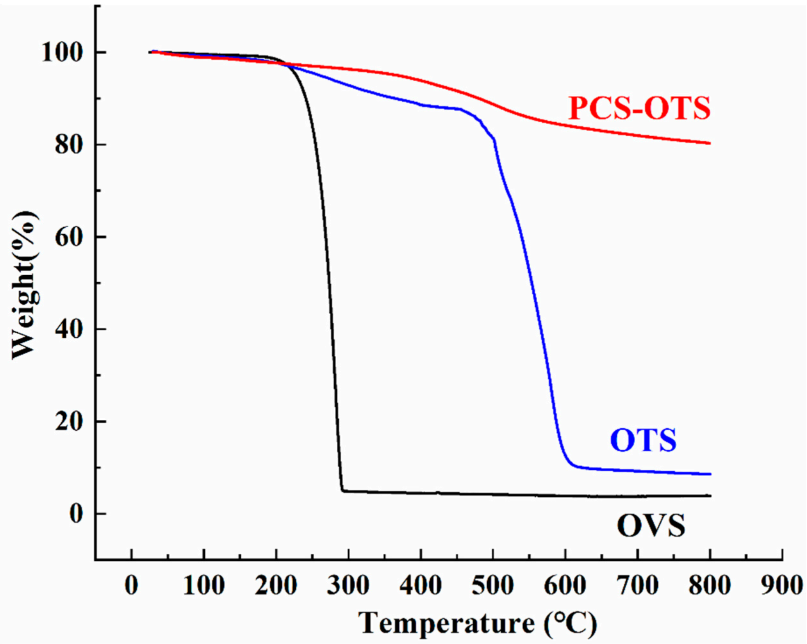

Figure S2. TGA curves of OVS, OTS and PCS-OTS in N_2.

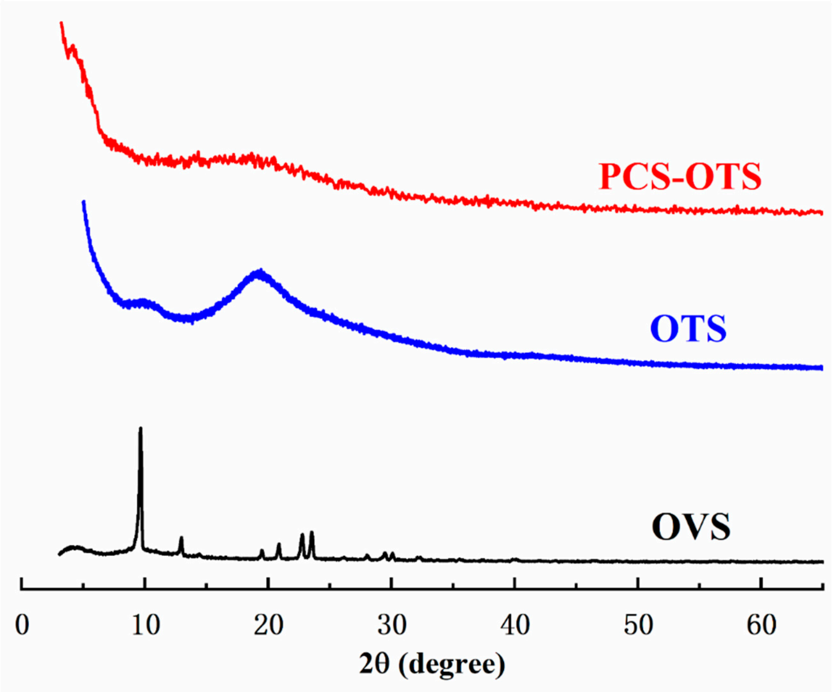

Figure S3. Powder XRD patterns of PCS-OTS, OTS and OVS.

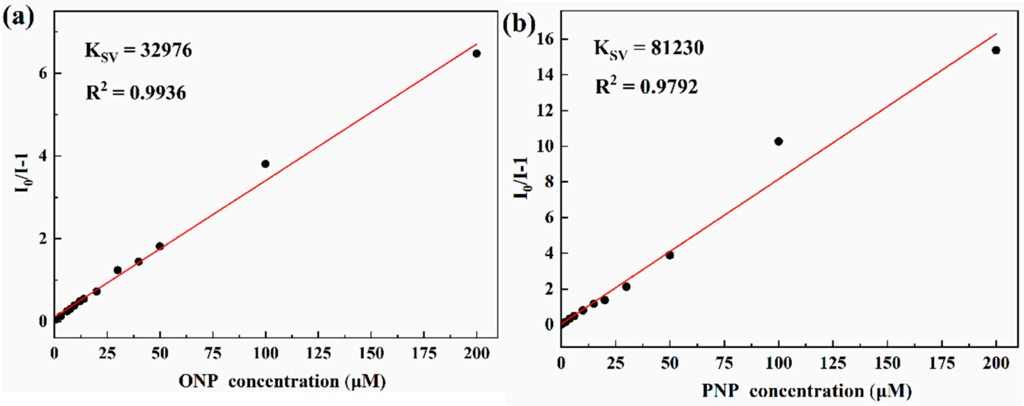

Figure S4. Stern-Volmer plot of $I_0/I-1$ of PCS-OTS versus [ONP] (**a**) and [PNP] (**b**) and K_{SV} value.

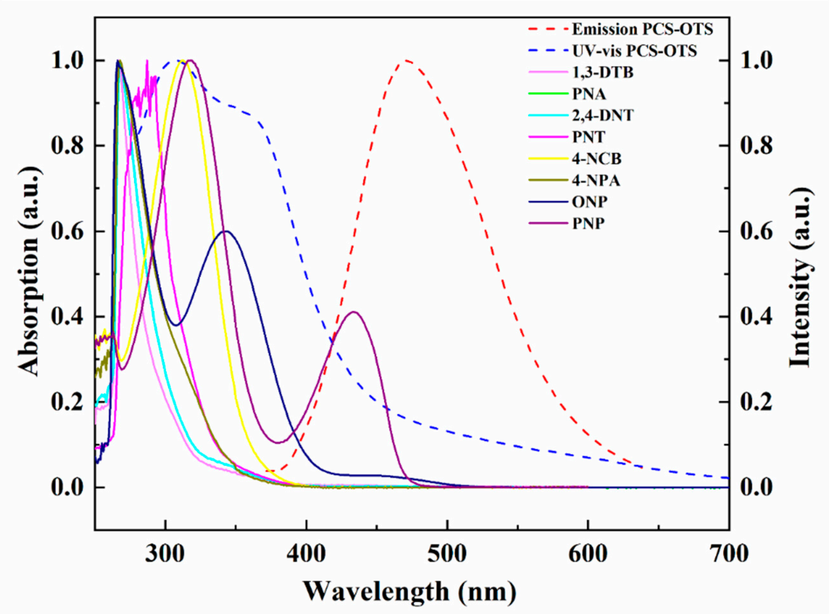

Figure S5. UV-vis absorption spectra of various analytes in DMF, and UV-vis and emission spectra of PCS-OTS in DMF suspension.

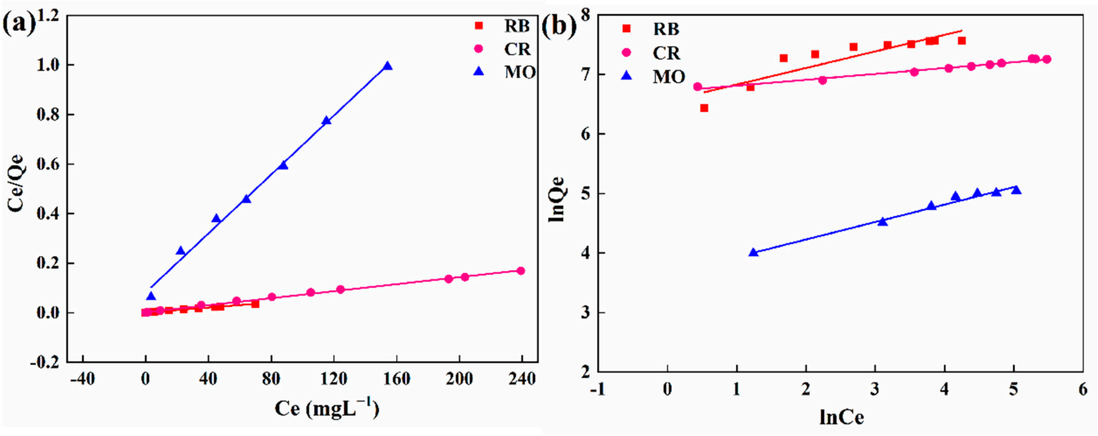

Figure S6. The Langmuir (**a**) and Freundlich (**b**) isotherm models for dyes solution onto PCS-OTS.

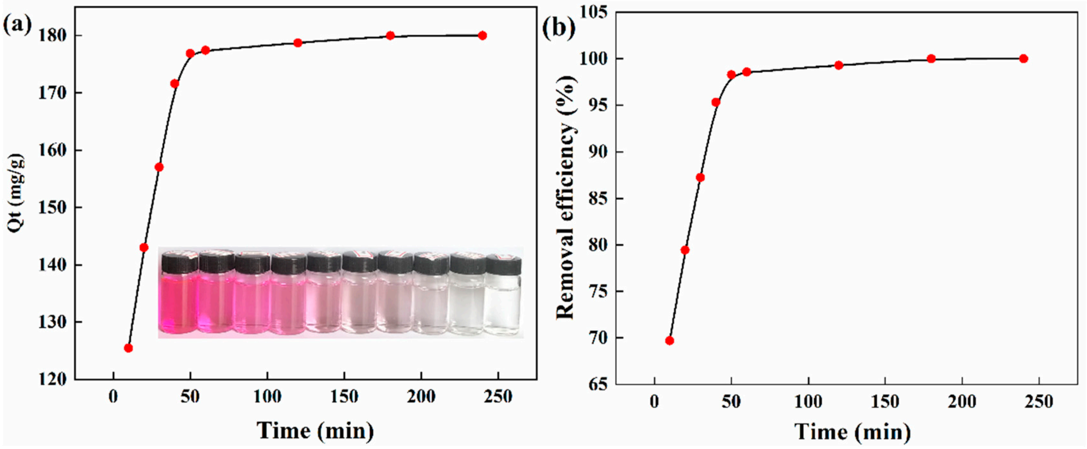

Figure S7. (**a**) Effect of contact time on the adsorption of Rhodamine B by PCS-OTS (inset is the photo of the dyes at 0 and 240 min). (**b**) Removal efficiency of Rhodamine B on PCS-OTS.

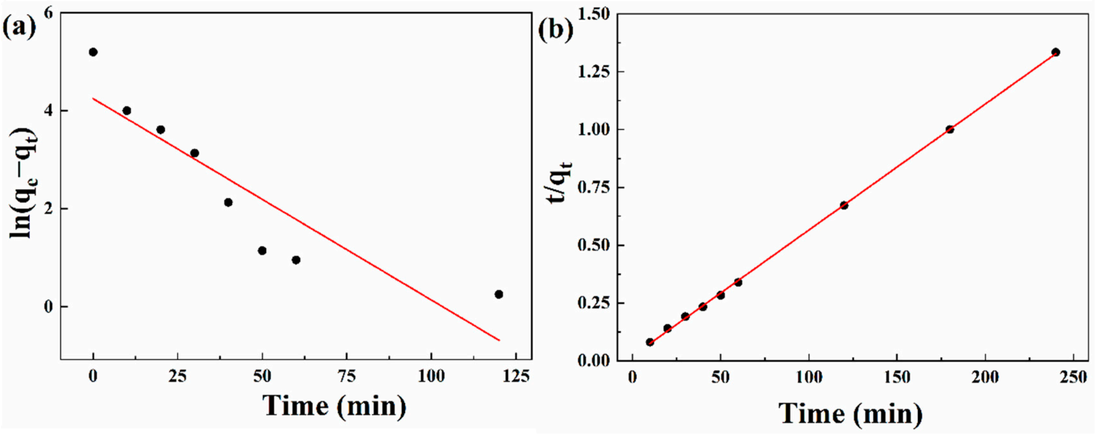

Figure S8. The pseudo-frist-order (**a**) and pseudo-second-order (**b**) kinetic model plots for RB adsorption by the PCS-OTS.

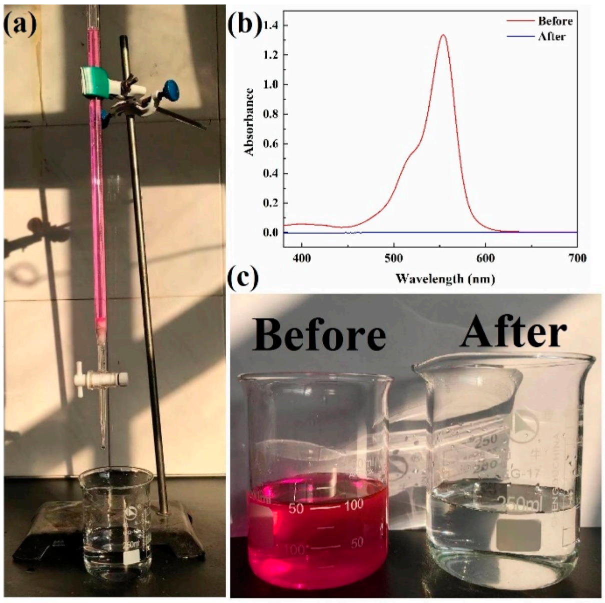

Figure S9. Process for the purification of the simulated wastewater.

Reference.

1. Wang, Q.; Liu, H.; Jiang, C.; Liu, H. Silsesquioxane-based triphenylamine functionalized porous polymer for CO_2, I_2 capture and nitro-aromatics detection. *Polymer* **2020**, *186*, 122004.
2. He, H.; Li, B.; Dong. J.; Lei. Y.; Wang, T.; Yu, Q.; Feng, Y.; Sun, Y. Mesostructured Nanomagnetic Polyhedral Oligomeric Silsesquioxanes (POSS) Incorporated with Dithiol Organic Anchors for Multiple Pollutants Capturing in Wastewater. *ACS Appl. Mater. Interfaces* **2013**, 5, 8058–8066.
3. Zhuang, X.; Wan, Y.; Feng, C.; Shen, Y.; Zhao, D. Highly Efficient Adsorption of Bulky Dye Molecules in Wastewater on Ordered Mesoporous Carbons. *Chem. Mater.* **2009**, *21*, 706–716.
4. Shen, R.; Liu, H. Construction of bimodal silsesquioxane-based porous materials from triphenylphosphine or triphenylphosphine oxide and their size-selective absorption for dye molecules. *RSC Adv.* **2016**, *6*, 37731–37739.
5. Zhao, J.; Ren, W.; Cheng, H., Graphene sponge for efficient and repeatable adsorption and desorption of water contaminations. *J. Mater. Chem.* **2012**, *22*, 20197–20202.
6. Shi, W.; Tao, S.; Yu, Y.; Wang, Y.; Ma, W. High performance adsorbents based on hierarchically porous silica for purifying multicomponent wastewater. *J. Mater. Chem.* **2011**, *21*, 15567–15574.
7. Ge, M.; Liu, H. A silsesquioxane-based thiophene-bridged hybrid nanoporous network as a highly efficient adsorbent for wastewater treatment. *J. Mater. Chem. A* **2016**, *4*, 16714–16722.
8. Liu H.; Liu H. Selective Dye Adsorption and Metal Ion Detection Using Multifunctional Silsesquioxane-Based Tetraphenylethene-Linked Nanoporous Polymers. *J. Mater. Chem. A* **2017**, *5*, 9156–9162.
9. Yang, R.; Wang, T.; Deng, W. Extraordinary Capability for Water Treatment Achieved by a Perfluorous Conjugated Microporous Polymer. *Sci.Rep*. **2015**, *5*, 10155.
10. Yang, S.; Wang, L.; Zhang, X.; Yang, W.; Song, G. Enhanced adsorption of Congo red dye by functionalized carbon nanotube/mixed metal oxides nanocomposites derived from layered double hydroxide precursor. *Chem. Eng. J.* **2015**, *275*, 315–321.

11. Wang, B.; Wu, H.; Yu, L.; Xu, R.; Lim, T.; Lou, X. Template-free Formation of Uniform Urchin-like α-FeOOH Hollow Spheres with Superior Capability for Water Treatment. *Adv. Mater.* **2012**, *24*, 1111–1116.
12. Sohn, K.; Joo, N.; Chang, H.; Roh, K.; Dong, J.; Huang, J. Oil absorbing graphene capsules by capillary molding. *Chem. Commun*. **2012**, *48*, 5968–5970.
13. Lei, W.; Portehault, D.; Liu, D.; Qin, S.; Chen Y. Porous boron nitride nanosheets for effective water cleaning. *Nat. Commun*. **2013**, *4*, 1777.
